# Identification of INHBA as a potential biomarker for gastric cancer through a comprehensive analysis

**DOI:** 10.1038/s41598-023-39784-1

**Published:** 2023-08-01

**Authors:** Fang Liu, Jiayi Liu, Xinrui Shi, Xiaojie Hu, Lai Wei, Bingjie Huo, Liang Chang, Yaqing Han, Guangjie Liu, Lei Yang

**Affiliations:** 1 Department of Thoracic Surgery, Hebei Medical University Fourth Affiliated Hospital, Shijiazhuang, 050001 Hebei People’s Republic of China; 2 Department of Epidemiology and Statistics, School of Public Health, Hebei Key Laboratory of Environment and Human Health, Hebei Medical University, Shijiazhuang, 050017 People’s Republic of China; 3 Department of General Surgery, Hebei Provincial People’s Hospital, Shijiazhuang, 050055 Hebei People’s Republic of China; 4 Department of Chinese Medicine, Hebei Medical University Fourth Affiliated Hospital, Shijiazhuang, 050001 Hebei People’s Republic of China; 5 Department of Pathology, Hebei Medical University Fourth Affiliated Hospital, Shijiazhuang, 050001 Hebei People’s Republic of China

**Keywords:** Cancer, Cancer genomics, Tumour immunology

## Abstract

Inhibin subunit beta A (INHBA) is a member of the transforming growth factor-beta (TGF-β) superfamily that plays a fundamental role in various cancers. However, a systematic analysis of the exact role of INHBA in patients with gastric cancer (GC) has not yet been conducted. We evaluated the expression levels of INHBA and the correlation between INHBA and GC prognosis in GC. The relationship between INHBA expression, immune infiltration levels, and type markers of immune cells in GC was also explored. In addition, we studied INHBA mutations, promoter methylation, and functional enrichment analysis. Besides, high expression levels of INHBA in GC were significantly related to unfavorable prognosis. INHBA was negatively correlated with B cell infiltration, but positively correlated with macrophage and most anticancer immunity steps. INHBA expression was positively correlated with the type markers of CD8+ T cells, neutrophils, macrophages, and dendritic cells. INHBA has a weak significant methylation level change between tumor and normal tissues and mainly enriched in cancer-related signaling pathways. The present study implies that INHBA may serve as a potential biomarker for predicting the prognosis of patients with GC. INHBA is a promising predictor of immunotherapy response, with higher levels of INHBA indicating greater sensitivity.

## Introduction

Owing to the development of techniques for the diagnosis and treatment of gastric cancer (GC), its incidence has decreased significantly; yet, it still remains one of the most prevalent malignancies and the third leading cause of cancer-related mortality worldwide^1,2^. Most GCs are induced by a complex interaction between epigenetic changes and environmental factors, such as *Helicobacter pylori* infection and trace element concentration^3–5^. At present, surgery and chemotherapy are the main therapeutics for patients with GC; however, the 5-year survival rates remain disappointing because many patients are still diagnosed for the first time at an advanced stage and relapse after treatment^6–8^. Therefore, more efforts should be made to identify beneficial biomarkers for early diagnosis and targeted therapy.

Inhibin subunit beta A (INHBA) is a member of the transforming growth factor-beta (TGF-β) superfamily that exerts a variety of biological functions, including immune response, sex determination, stem cell differentiation, and control of cellular migration and proliferation^9–12^. Emerging studies have shown that INHBA is aberrantly expressed in multiple tumor types, such as nasopharyngeal carcinoma^13^, lung adenocarcinoma (LUAD)^14^, ovarian cancer^15,16^, colon cancer^17,18^, esophageal squamous cell carcinoma^19^, pancreatic cancer^20^, breast cancer^21^ and bladder cancer^22^ and serves as a prognostic factor in these conditions. Although Wang et al. showed that INHBA is highly expressed in GC tumor tissues and is a prognostic biomarker for patients with GC^23,24^, a systematic analysis of the exact role of INHBA in patients with GC has not been conducted, which is necessary to reveal the underlying mechanisms of INHBA in GC.

The present study aimed to systematically assess the correlation between INHBA expression and GC survival, as well as, the function and mechanism of action of INHBA. Typically, INHBA mRNA expression was detected in both GC and normal tissues using the Tumor Immune Estimation Resource (TIMER), Gene Expression Profiling Interactive Analysis (GEPIA2), Gene Expression Omnibus (GEO), Oncomine, and UALCAN databases. The significance of INHBA in predicting the prognosis of GC was analyzed based on the Kaplan–Meier plotter database. The relationship between INHBA expression and immune infiltration in GC was explored using the TIMER database. With help of the MEXPRESS database, we explored whether INHBA expression is correlated with changes in INHBA methylation in GC samples, as compared to that in normal samples. In addition, Gene Ontology (GO) and Kyoto Encyclopedia of Genes and Genomes (KEGG) analyses were used to examine the underlying mechanism of action. The findings of this study shed light on the important role of INHBA in GC and illustrate the potential mechanisms related to immune infiltration in GC. They also provide a theoretical foundation for early diagnosis, prognostic evaluation, and specific treatment of GC.

## Results

### INHBA expression in patients with GC

**Figure 1:**
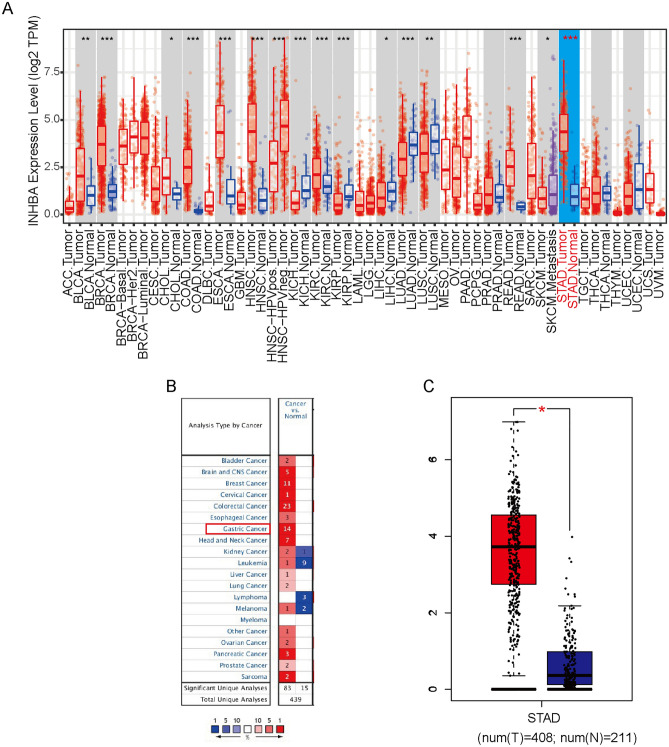
INHBA expression levels in different human cancers. (**A**) The Diff Exp module was used to analyze the expression of INHBA in all tumor samples and normal tissues in TIMER database. https://cistrome.shinyapps.io/timer/. (**B**) Change in INHBA expression levels in datasets of different cancers, as compared to those in normal tissues, as determined using Oncomine database. (**C**) The expression level of INHBA in GC tumor tissues and paired normal tissues, as determined using GEPIA database. *P < 0.05; **P < 0.01; ***P < 0.001, and *NS*: *P* > 0.05. http://gepia2.cancer-pku.cn/#general.

A comparison of INHBA mRNA expression in various cancer types and normal tissues in the TIMER database revealed significantly higher INHBA expression in bladder urothelial carcinoma, breast invasive carcinoma, cholangiocarcinoma , colon adenocarcinoma, esophageal carcinoma, head and neck squamous cell carcinoma , kidney chromophobe, kidney renal clear cell carcinoma, rectum adenocarcinoma, and stomach adenocarcinoma tissues. In contrast, INHBA expression was significantly lower in kidney renal papillary cell carcinoma, lung adenocarcinoma, and lung squamous cell carcinoma tissues than in adjacent normal tissues (Fig. 1A). We then compared the expression levels of INHBA in GC tumor tissues with those in normal tissues using the Oncomine and GEPIA databases. The results revealed that INHBA expression was significantly higher in GC tissues than in normal tissues (*P* < 0.05) (Fig. 1B,C).

**Figure 2:**
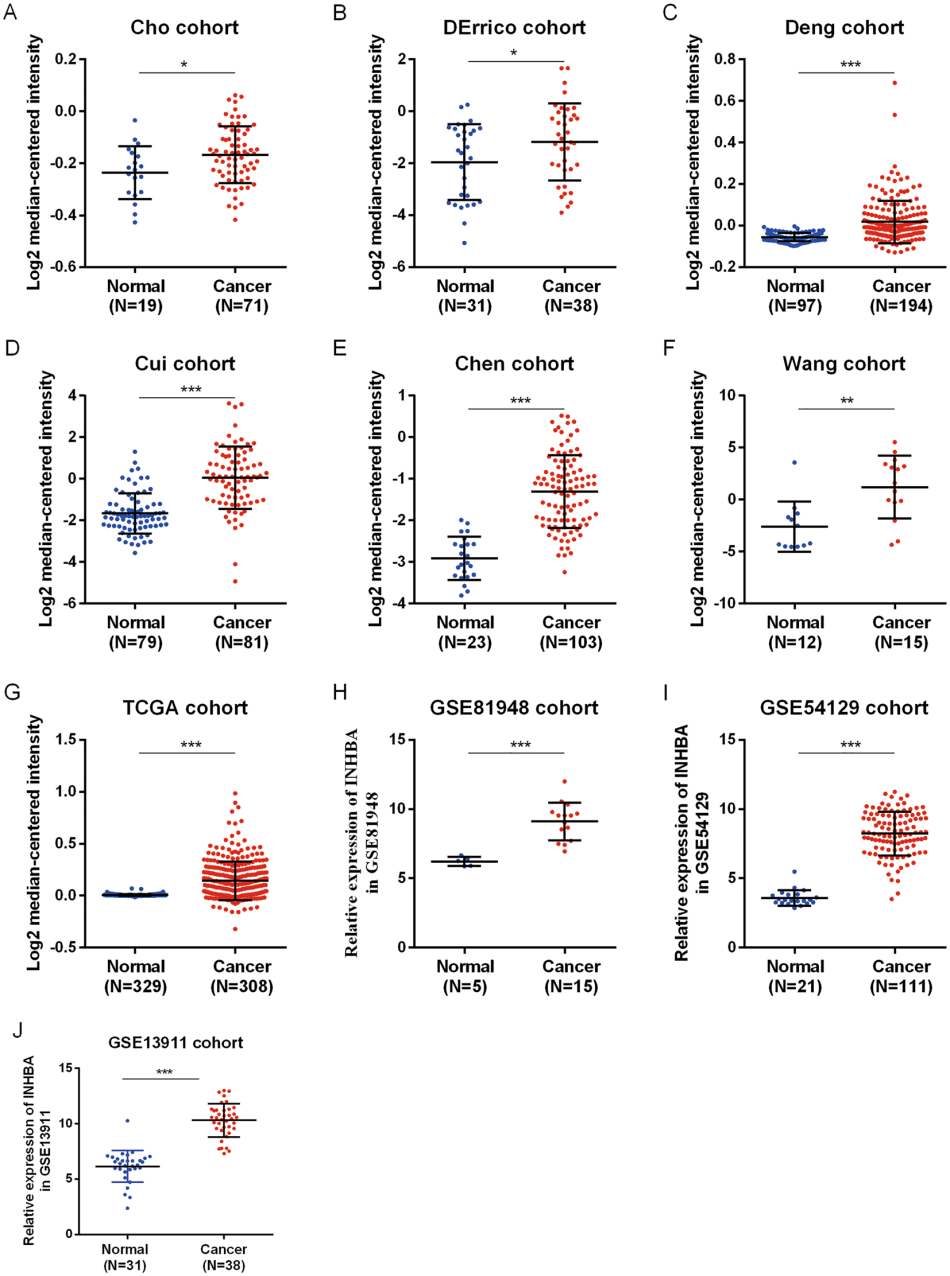
Relative expression levels of INHBA in the GC tissues, as compared to those in non-cancerous tissues, in ten cohorts, as determined using Oncomine and GEO databases. *P < 0.05; **P < 0.01, and ***P < 0.001. (**A**–**G**) Oncomine database. (**H**–**J**) GEO database. https://www.ncbi.nlm.nih.gov/geo/tools/profileGraph.cgi?ID=GDS1210:X57579_s_at.

**Figure 3:**
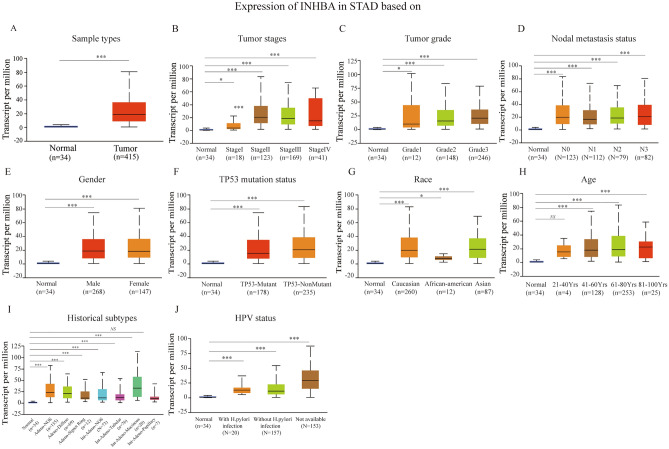
UALCAN database was used to evaluate the expression of INHBA in different tumor subgroups. Boxplot showed the relative expression level of INHBA in the subgroup of patients with gastric cancer (UALCAN). https://ualcan.path.uab.edu/cgi-bin/Pan-cancer-CPTAC.pl?genenam=INHBA (**A**) Comparison of the transcriptional expression level of INHBA between gastric cancer (GC) tissues and non-cancerous tissues. https://ualcan.path.uab.edu/cgi-bin/Pan-cancer-CPTAC.pl?genenam=INHBA (**B**–**J**) Boxplot showing correlation of tumor stage, grade, nodal metastasis status, gender, TP53 mutation status, race, age, historical subtype, and HPV status with INHBA expression in GC. (N0: No regional lymph node metastasis; N1: metastases in 1 to 3 axillary lymph nodes; N2: metastases in 4 to 9 axillary lymph nodes; N3: metastases in 10 or more axillary lymph nodes. *P < 0.05; **P < 0.01; ***P < 0.001, and *NS*: *P* > 0.05.

**Figure 4:**
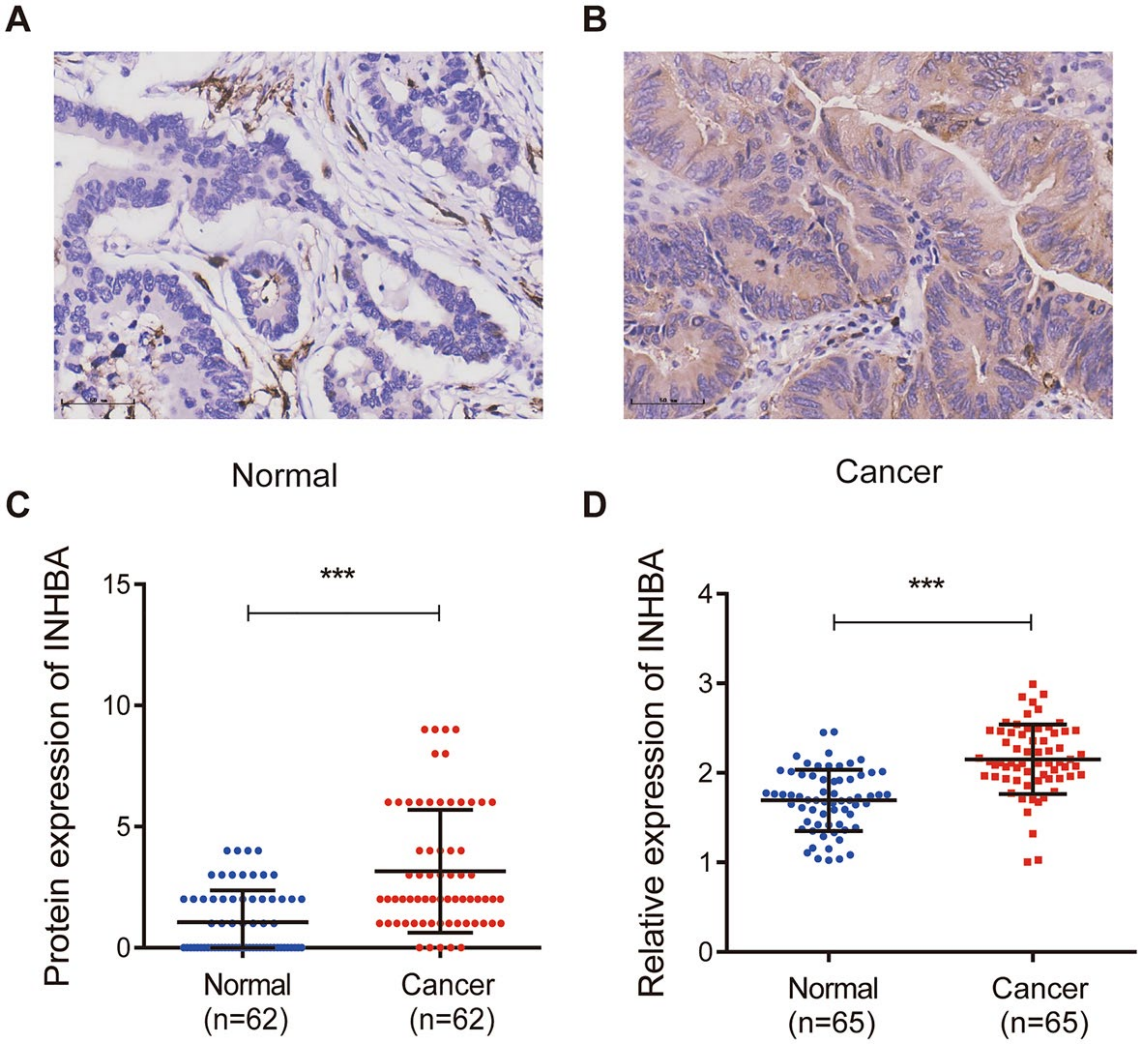
The Oncomine database was used to determine protein and mRNA expression in pairs of gastric and normal gastric tissues. (**A**–**C**) Comparison of INHBA protein expression in 62 paired normal gastric tissues and gastric cancer tissues. (**D**) Relative mRNA expression of INHBA in 65 paired normal gastric tissues and cancer tissues. ***P < 0.001.

When the expression level of INHBA was analyzed in Cho GC, DErrico GC, Deng GC, Cui GC, Chen GC, TCGA GC, Wang GC, GSE81948, GSE54129, and GSE13911 datasets, the scatter plot showed that the expression level of INHBA was fundamentally upregulated in GC patient tumor tissues, as compared to that in adjacent normal tissues (Fig. 2). Previous studies have confirmed using IHC and western blot that INHBA protein is highly expressed in GC tumor tissues^23,25,26^. Further analysis of diverse clinicopathological characteristics of 415 GC samples in the UALCAN database indicated higher transcriptional levels of INHBA. In the stage subgroup (normal-vs-Stage I, normal-vs-Stage II, normal-vs-Stage III, and normal-vs-Stage IV), tumor grade subtype (normal-vs-grade 1, normal-vs-grade 2, and normal-vs-grade 3), nodal metastasis status subgroup (normal-vs-N0, normal-vs-N1, normal-vs-N2, and normal-vs-N3), gender subgroup (normal-vs-male and normal-vs-female), TP53 mutation status subgroup (normal-vs-TP53 mutant and normal-vs-non-mutant), race subgroup (normal-vs-Caucasian, normal-vs-African American, and normal-vs-Asian), HPV status subgroup (normal-vs-with HPV infection status and normal-vs-without HPV infection status) analyses, as well as age subgroup analysis, INHBA expression was fundamentally higher in GC patients (Fig. 3). These findings suggested that INHBA expression can serve as a potential diagnostic biomarker for GC. We validated the protein and mRNA expression of INHBA in GC tissues and found that INHBA was significantly upregulated in GC tissues, as compared to that in normal tissues (Fig. 4).

### Diagnostic value of INHBA in patients with GC

**Figure 5:**
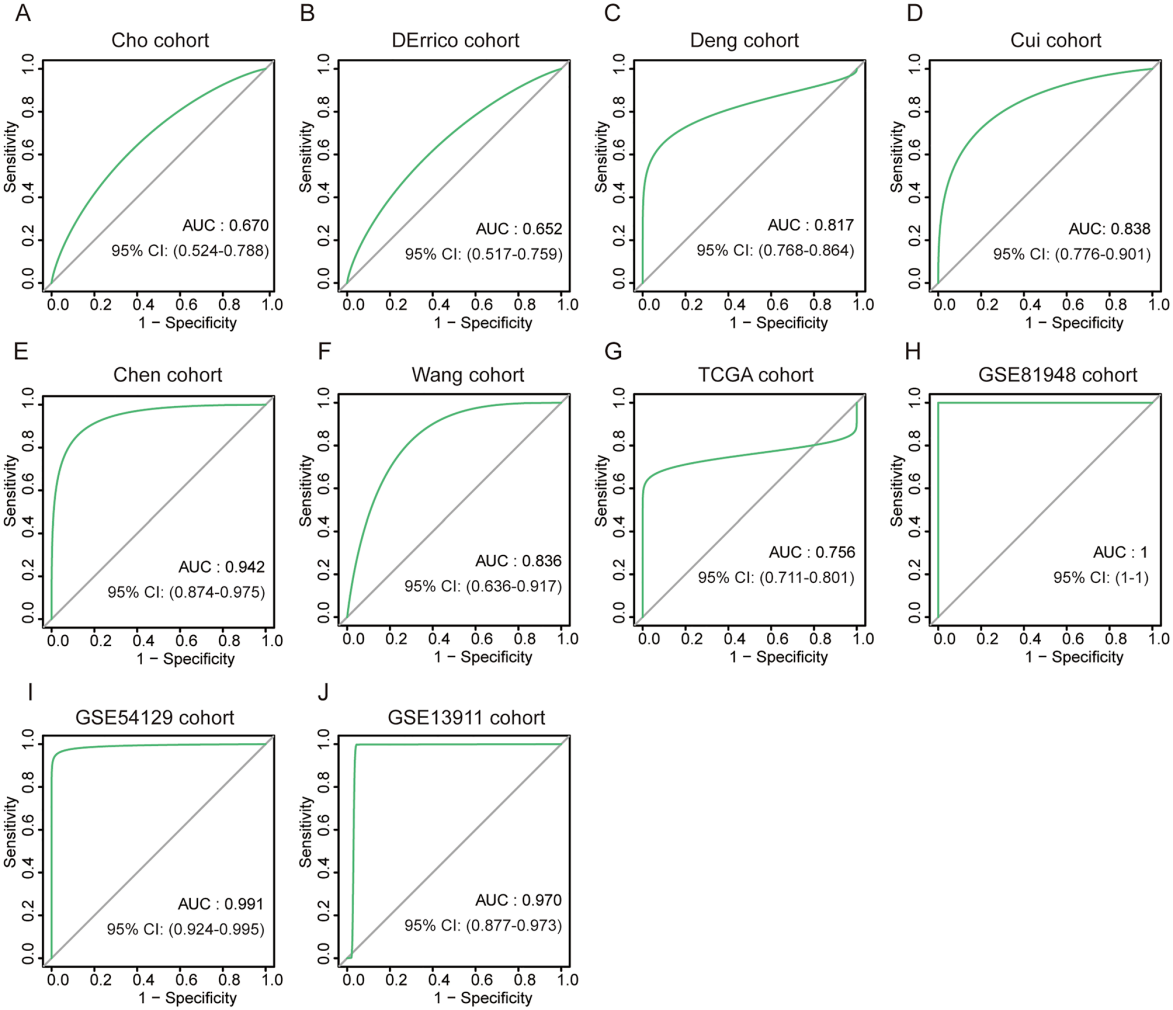
Based on the data of Cho GC, DErrico GC, Deng GC, Cui GC, Chen GC, TCGA GC, Wang GC, GSE81948 https://www.ncbi.nlm.nih.gov/geo/query/acc.cgi?acc=GSE81948, GSE54129 https://www.ncbi.nlm.nih.gov/geo/query/acc.cgi?acc=GSE54129, GSE13911 https://www.ncbi.nlm.nih.gov/geo/query/acc.cgi?acc=GSE13911 data sets, using the pROC package in R software, ROC curves of INHBA were drawn to distinguish GC patients from healthy individuals.

Based on the difference in INHBA expression in GC, we further explored the diagnostic value of INHBA for distinguishing GC patients from healthy individuals, by generating ROC curves based on the data from Cho GC, DErrico GC, Deng GC, Cui GC, Chen GC, TCGA GC, Wang GC, GSE81948, GSE54129, and GSE13911 datasets (Fig. 5). The results showed that INHBA had high diagnostic value for distinguishing GC patients from healthy individuals (Cho GC, AUC = 0.670, 95% CI 0.524–0.788], Fig. 5A; DErrico GC, AUC = 0.652, 95% CI 0.517–0.759, Fig. 5B; Deng GC, AUC = 0.817, 95% CI 0.768–0.864, Fig. 5C; Cui GC, AUC = 0.838, 95% CI 0.776–0.901, Fig. 5D; Chen GC, AUC = 0.942, 95% CI 0.874–0.975, Fig. 5E; Wang GC, AUC = 0.836, 95% CI 0.636–0.917, Fig. 5F; TCGA GC, AUC = 0.756, 95% CI 0.711–0.801, Fig. 5G; GSE81948 GC, AUC = 1, 95% CI 1–1, Fig. 5H; GSE54129 GC, AUC = 0.991, 95% CI 0.924–0.996, Fig. 5I; GSE13911 GC, AUC = 0.970, 95% CI 0.877–0.973, Fig. 5J).

### Correlation between the expression level of INHBA and prognosis in patients with GC

**Figure 6:**
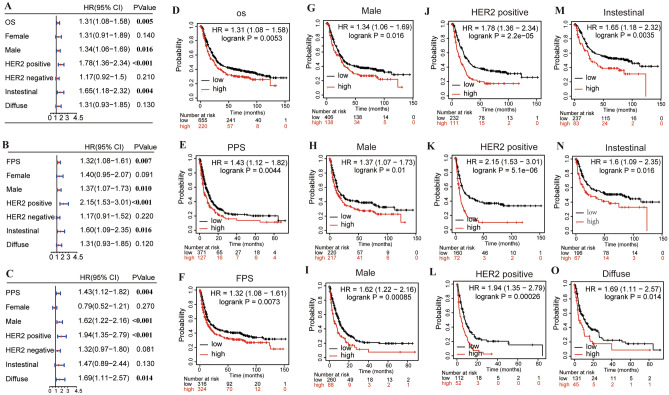
Correlation of INHBA expression with survival analysis in gastric cancer. Kaplan–Meier plotter was applied to evaluate the prognostic value of INHBA. Survival analysis in patients within gender, HER2 status, and Lauren classification subgroups. (**A**–**F**) Correlation of INHBA expression with overall survival (OS), first progression survival (FPS), and post-progression survival (PPS). (**G**–**I**) In terms of sex subtypes, the correlation of INHBA expression with OS, FPS and PPS. (**J**–**L**) In terms of HER2 status subtypes, the correlation of INHBA expression with OS, FPS and PPS. (**M**–**O**) In terms of Lauren classification subtypes, the correlation of INHBA expression with OS, FPS and PPS. https://kmplot.com/analysis/index.php?p=service.

Based on the difference in INHBA expression in GC, we further analyzed the correlation between INHBA expression and prognosis in patients with GC to ascertain whether INHBA acts as a promoter or suppressor of GC. The Kaplan–Meier plotter was used to investigate the relationship between INHBA expression and prognosis in patients with GC, including OS, FPS, and PPS. The results showed that high expression of INHBA in GC was significantly related to worse OS (HR = 1.31; 95% CI 1.08–1.58; *P* = 0.015), FPS (HR = 1.32; 95% CI 1.08–1.61; *P* = 0.007), and PPS (1.43; 95% CI 1.12–1.82; *P* = 0.004), as shown in Fig. 6A–F. We further analyzed the correlation between the expression levels of INHBA and clinicopathological subtypes, such as sex, HER2 status, and Lauren classification. With respect to sex subtypes, high expression of INHBA in male patients with GC was associated with poor OS (HR = 1.34; 95% CI 1.06–1.69; *P* = 0.016), FPS (HR = 1.37; 95% CI 1.07–1.73; *P* = 0.010), and PPS (HR = 1.62; 95% CI 1.22–2.16; *P* = 0.00085) as shown in Fig. 6G–I. In HER2 status subtypes, high expression of INHBA in HER2-positive patients with GC was associated with worse OS (HR = 1.78; 95% CI 1.36–2.34; *P* < 0.011), FPS (HR = 2.15; 95% CI 1.53–3.01; *P* < 0.011), and PPS (HR = 1.94; 95% CI 1.35–2.79; *P* < 0.011), as shown in Fig. 6J–L. In the Lauren classification subtypes, high expression of INHBA in intestinal patients with GC had worse OS in Fig. 6M,N (HR = 1.65; 95% CI 1.18–2.32; *P* = 0.004) and FPS (HR = 1.60; 95% CI 1.09–2.35; *P* = 0.016); however, Fig. 6O indicated that the high expression of INHBA in diffuse patients with GC had worse PPS (HR = 1.69; 95% CI 1.11–2.57; *P* = 0.014) . These results suggested that INHBA may serve as a potential biomarker for specific GC subtypes.

### Correlation of immune infiltrates with INHBA in GC

**Figure 7:**
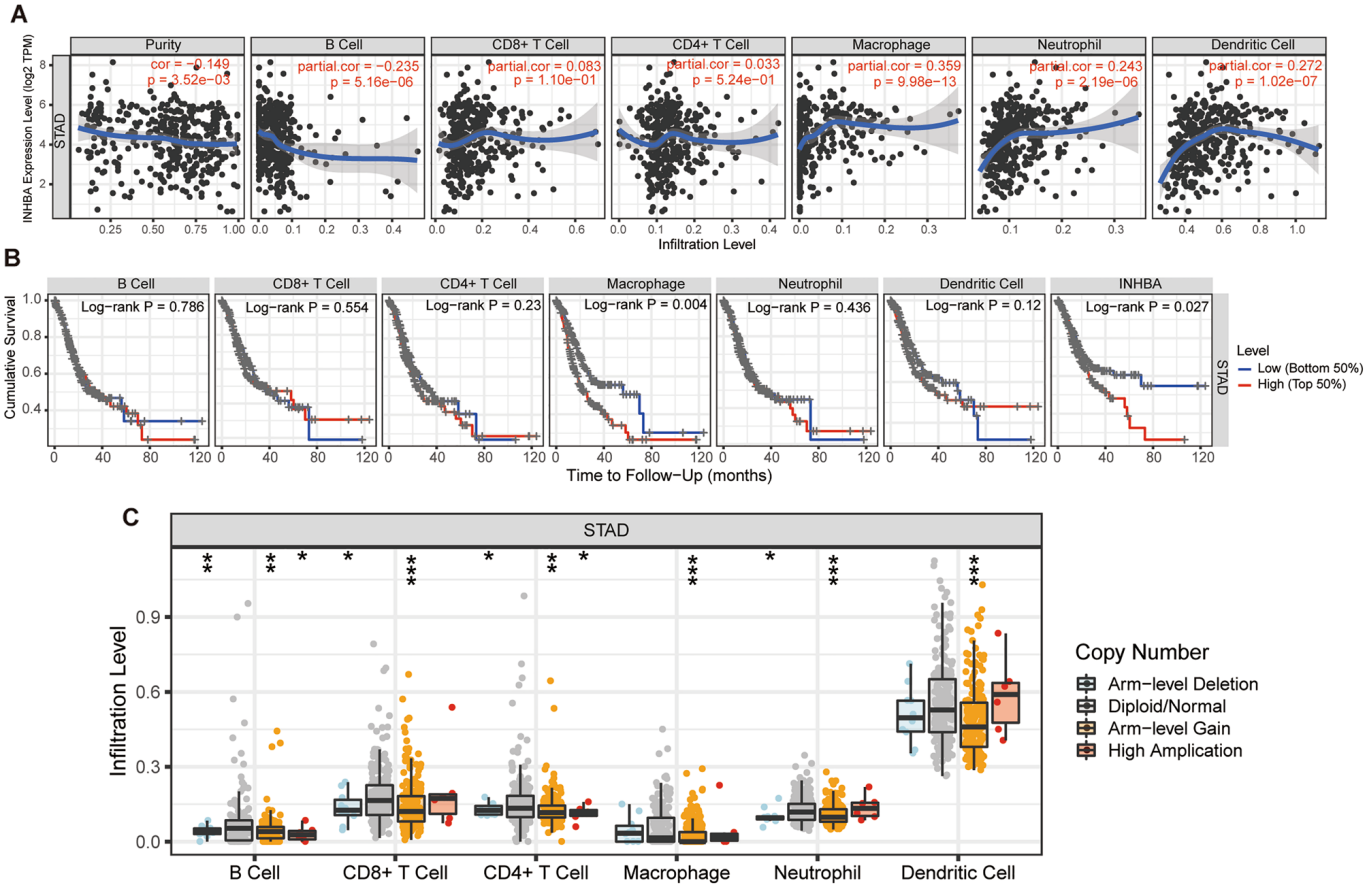
Correlation between immune infiltrates and INHBA expression in B cells, CD4+ T cells, CD8+ T cells, neutrophils, macrophages, and dendritic cells of GC (TIMER). (**A**) Correlation between INHBA expression and abundance of immune infiltrates. (**B**) Clinical outcomes and abundance of immune infiltrates of INHBA expression. (**C**) Correlation between abundance of immune infiltrates and somatic copy number alterations of INHBA in GC. *P < 0.05; **P < 0.01, and ***P < 0.001. https://cistrome.shinyapps.io/timer/.

Previous studies have shown that tumor infiltration is significantly associated with the progression and prognosis of GC^27–29^. Therefore, we used the TIMER database to investigate whether the expression levels of INHBA in GC tumors were correlated with immune infiltration. The results showed that INHBA was negatively correlated with B cells and positively correlated with macrophage, neutrophil, and dendritic cell infiltration (*P* < 0.05; Fig. 7A). Cumulative survival analysis revealed that macrophage immune infiltrates were significantly associated (*P* < 0.05) with INHBA in GC, indicating that macrophages negatively affect prognosis; this finding warrants further investigation (Fig. 7B). Finally, somatic copy number alterations, including deep deletion (-2), arm-level deletion (-1), diploid/normal (0), arm-level gain (1), and high amplification (2), were characterized using GISTIC 2.0. Box plots have been generated to show the distribution of each immune subset at each copy number status of INHBA in GC (Fig. 7C).

### Relationship between INHBA expression and immune cell type markers in GC

We analyzed the correlation between the expression of INHBA and different immune cells type markers in GC based on the TIMER database. The results showed that INHBA expression in GC was positively correlated with CD38 expression in B cells (Table 1). INHBA expression in GC was also positively correlated with CD8A expression in CD8+ T cells. Similarly, INHBA expression in GC was positively correlated with MPO, FCGR3B, FPR1, and CSF3R expression in neutrophils, and CD209 expression in dendritic cells (Table 1). INHBA expression in GC was positively correlated with CD68, CD84, CD163, and MS4A4A expression in macrophages (Table 1). These results further confirmed that INHBA expression in GC is correlated with immune infiltration.

**Table 1:** Correlation analysis between INHBA and immune cell type markers in the TCGA-STAD cohort obtained from TIMER database.

Cell type	Gene marker	None		Purity	
		Cor	*P*	Cor	*P*
B cells	CD19	−0.023	6.04E−01	− 0.041	4.28E−01
	FCRL2	0.021	6.66E−01	− 0.005	9.20E−01
	CD38	0.136	**5.00E−03**	0.091	7.56E−01
	MS4A1	− 0.021	6.74E−01	− 0.061	2.38E−01
CD8+ T cells	CD8A	0.120	**1.50E−02**	0.083	1.09E−01
	CD8B	0.037	4.51E−01	0.017	7.47E−01
Neutrophils	MPO	0.223	**4.50E−06**	0.215	**2.38E−05**
	FCGR3B	0.153	**1.73E−03**	0.130	**1.16E−02**
	FPR1	0.334	**5.68E−13**	0.328	**5.92E−11**
	CSF3R	0.287	**2.52E−09**	0.263	**1.94E−07**
	S100A12	0.064	1.96E−01	0.030	2.66 − 01
Macrophages	CD68	0.209	**1.76E−05**	0.196	**1.27E−04**
	CD84	0.208	**1.98E−05**	0.193	**1.54E−04**
	CD163	0.312	**8.29E−11**	0.281	**2.67E−08**
	MS4A4A	0.310	**1.10E−10**	0.292	**6.76E−09**
Dendritic cells	CD209	0.137	**9.14E−03**	0.101	**5.00E−02**
	CD1C	0.005	9.24E−01	− 0.018	8.01E−01

STAD, stomach adenocarcinoma; Cor, r value of Spearman’s correlation; purity, correlation adjusted by purity; values in bold have a level of significance of* P* < 0.05.

### Prognostic analysis of INHBA expression in GC, based on immune cells

**Figure 8:**
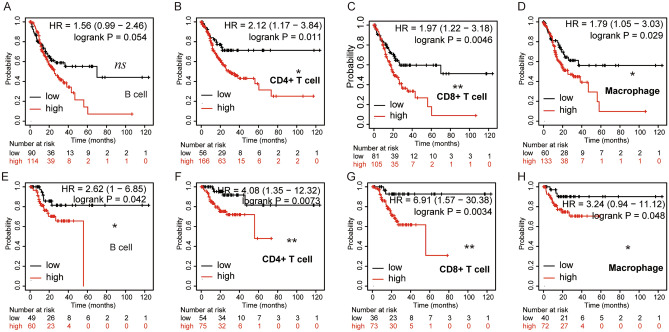
Comparison of Kaplan–Meier curves for high and low expression of INHBA in GC, based on immune cells subgroup. https://kmplot.com/analysis/index.php?p=service (**A**–**D**) Relationship between INHBA expression of different immune cell subgroups and overall survival in STAD. (**E**–**H**) Relationship between INHBA expression of different immune cell subgroups and relapse-free survival in STAD. *P < 0.05; **P < 0.01, and *NS*: *P* > 0.05.

We confirmed that INHBA expression was correlated with immune infiltration in GC, and that the expression of INHBA was also related to the poor prognosis of patients with GC. Thus, we speculated that the expression of INHBA in GC affected prognosis, partly due to immune infiltration. We then performed a prognostic analysis based on the expression levels of INHBA (in GC) in related immune cell subgroups using the Kaplan–Meier plotter database. The results revealed that the high expression of INHBA (in GC) in enriched CD4+ T cell cohorts (HR = 2.12; 95% CI 1.17–3.84; *P* = 0.011), CD8+ T cells (HR = 1.97; 95% CI 1.12–3.18; *P* = 0.0046), and macrophages (HR = 1.79; 95% CI 1.05–3.03; *P* = 0.029) had worse OS (Fig. 8B–D), while there was no statistical significance in case of the B cell cohort (Fig. 8A). The high expression of INHBA (in GC) in enriched B cell (HR = 2.62; 95% CI 1–6.85; *P* = 0.042), CD4+ T cell (HR = 4.08; 95% CI 1.35–12.32; *P* = 0.0073), CD8+ T cell (HR = 6.91; 95% CI 1.57–30.38; *P* = 0.0034), and macrophage (HR = 3.24; 95% CI 0.94–11.12; *P* = 0.048) cohorts had worse RFS (Fig. 8E–H). These results suggested that high INHBA expression in GC may affect prognosis, partly because of immune infiltration.

### Therapeutic roles of INHBA in GC

**Figure 9:**
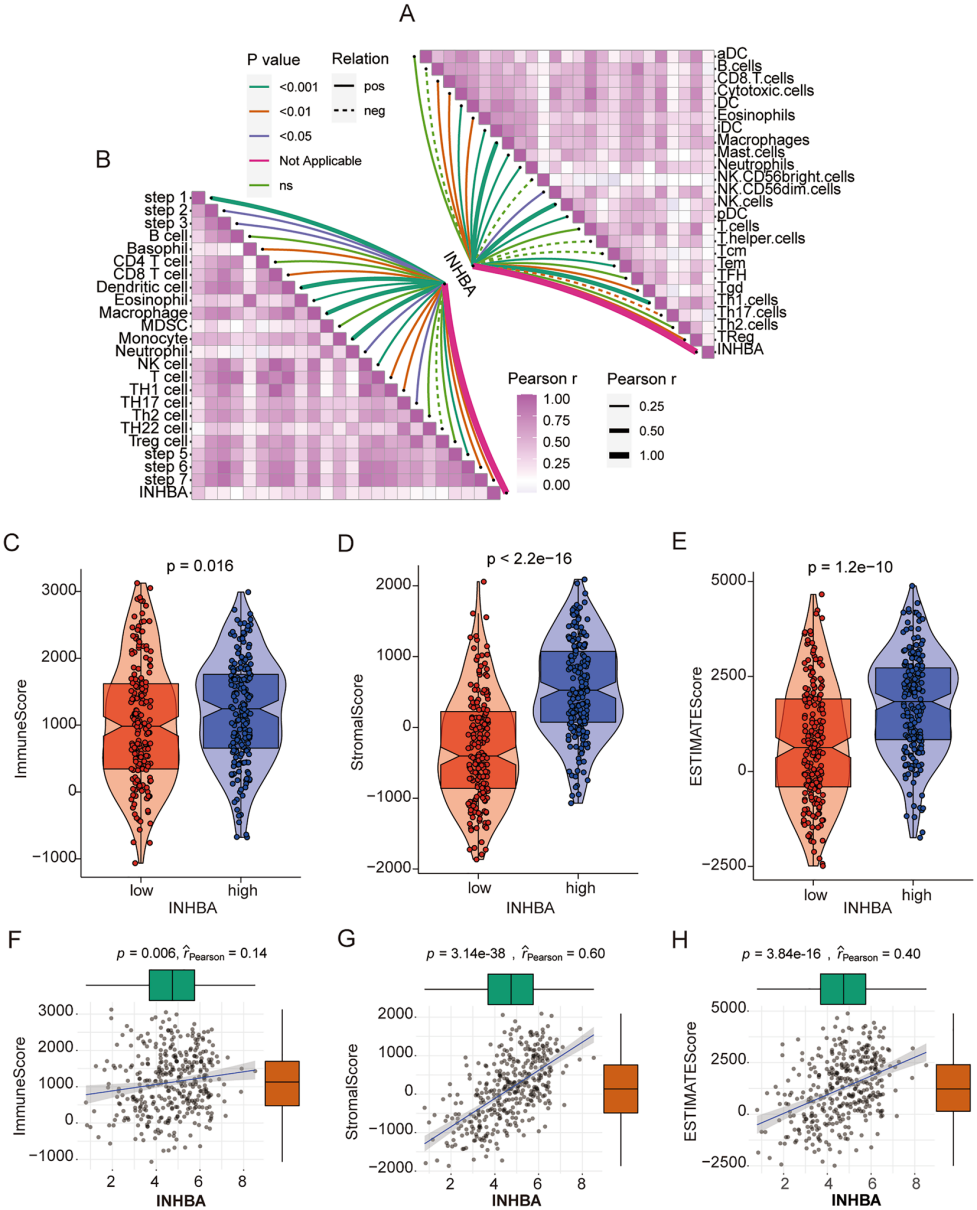
Correlation between INHBA expression and the TME in GC. (**A**) Correlation of INHBA expression and tumor-infiltrating immune cells. (**B**) Correlation of INHBA expression and the steps of the cancer immunity cycle. (**C**–**E**) Comparison of ImmuneScore, StromalScore, and ESTIMATEScore between high and low INHBA groups. (**F**–**H**) Correlation of INHBA expression and ImmuneScore, StromalScore, and ESTIMATEScore.

**Figure 10:**
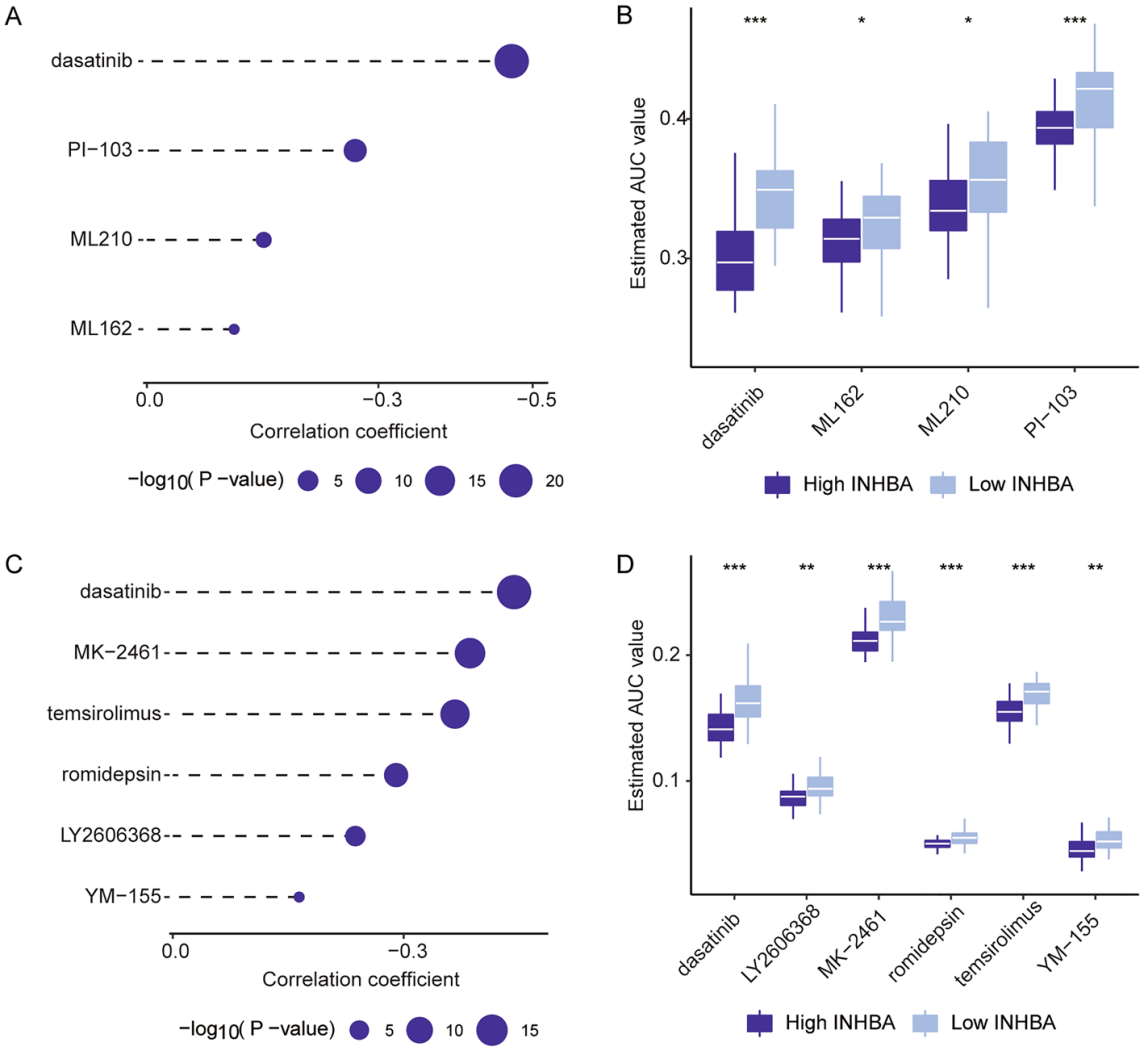
Potential therapeutic drugs for GC patients with high INHBA expression. (**A**–**B**) Results for Spearman’s correlation and differential drug-response analyses of four CTRP-obtained compounds. (**C**–**D**) Results for Spearman’s correlation and differential drug-response analyses of six PRISM-obtained compounds. Note: Lower estimated AUC indicates greater drug sensitivity. *P < 0.05; **P < 0.01, and ***P < 0.001.

Immune cell infiltration in GC was further explored using ssGSEA algorithms. The correlation between INHBA expression and immune cell infiltration was analyzed, which suggested that INHBA was significantly correlated with the TME and immune cell infiltration (Fig. 9A). Our research indicated that INHBA was significantly positively correlated with most steps of the cancer immune cycle (Fig. 9B). Moreover, the ImmuneScore, StromalScore, and ESTIMATEScore were higher in INHBA-high patients with GC than in INHBA-low patients with GC (Fig. 9C–H). Moreover, we used CTRP and PRISM to identify promising therapeutic drugs for patients with high INHBA levels. First, differential compound sensitivity analysis between the INHBA-high (top decile) and INHBA-low (bottom decile) groups was performed to identify drugs with lower estimated AUC values (log_2_ FC > 0.1). Following this, Spearman’s correlation analysis between INHBA and AUC value was conducted, to identify drugs with negative correlation coefficients (*r* < -0.2 for CTRP and PRISM, *P* < 0.05). Finally, we identified four CTRP-derived drugs (including dasatinib, PI-103, ML210, and ML162, Fig. 10A,B) and six PRISM-derived compounds (including dasatinib, mk-2461, temsirolimus, romidepsin, YM-155, and LY2606368, Fig. 10C,D). As indicated, the estimated AUC values of these drugs were negatively correlated with INHBA in GC.

### INHBA gene alterations in GC

**Figure 11:**
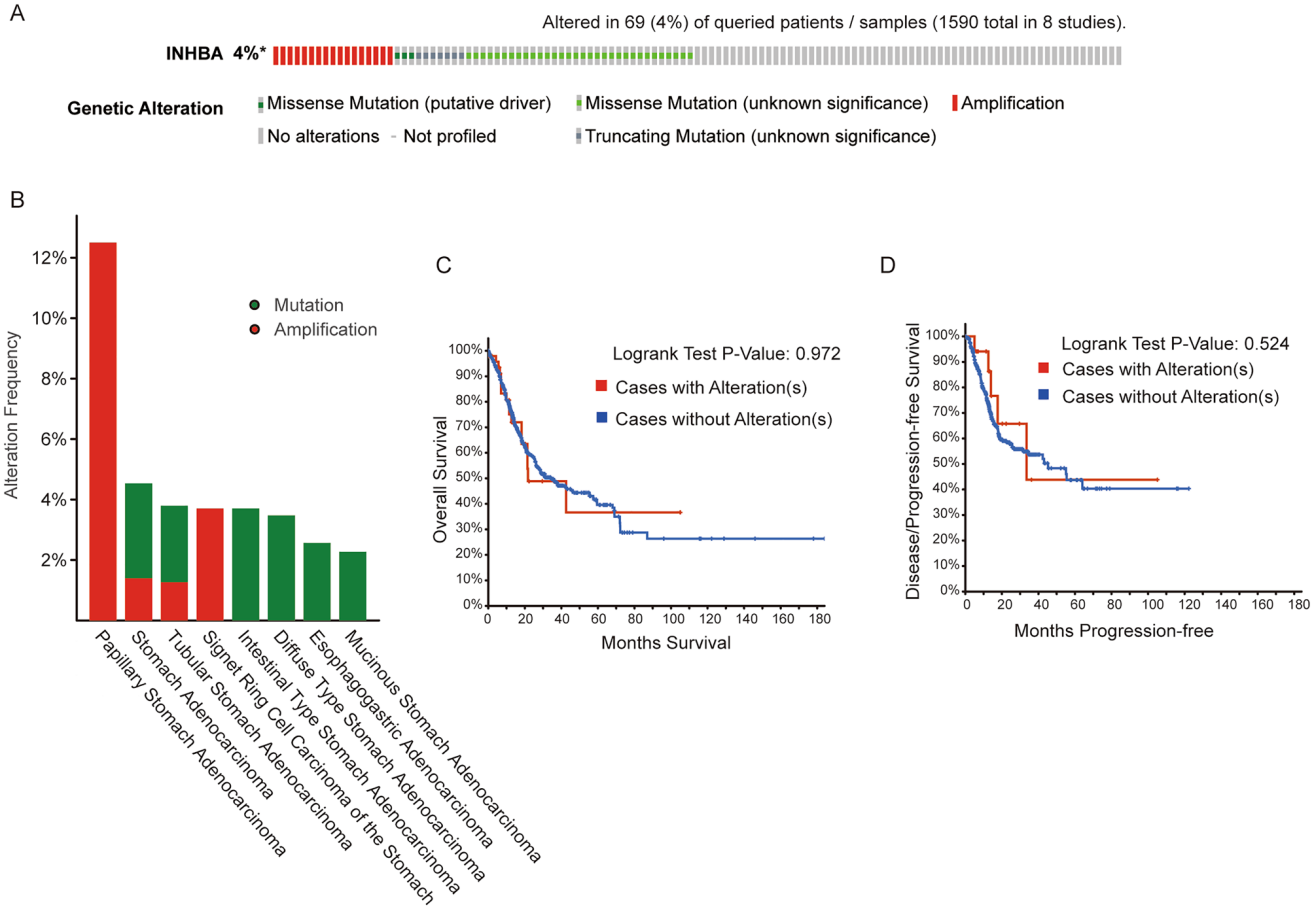
Visual summary of INHBA alterations in gastric cancer (Cbioportal databases and TCGA). (**A**) OncoPrint of INHBA genetic alterations in GC. (**B**) The genetic alteration type and frequency of INHBA were studied in various GC samples. (**C**) Kaplan–Meier analysis of the effect of INHBA disorder on overall survival. (**D**) Kaplan–Meier analysis of the effect of INHBA disorder on progression-free survival.

To explore the sequence alterations in INHBA in GC, we used the cBioPortal database to investigate the types and frequency of INHBA modifications (in GC) in the sequencing data of STAD patients obtained from TCGA. As shown in Fig. 11A, 69 of 1590 (4%) patients with GC showed alterations. Further studies suggested that mRNA upregulation and mutations are the most common types of INHBA alterations in patients with GC (Fig. 11B). In addition, the results of the Kaplan–Meier plotter and log-rank test demonstrated no significant statistical difference in OS and DFS in cases with and without INHBA alterations (*P* = 0.972 and *P* = 0.524, respectively; Fig. 11C,D).

### Correlation between INHBA expression and methylation around the promoter region

**Figure 12:**
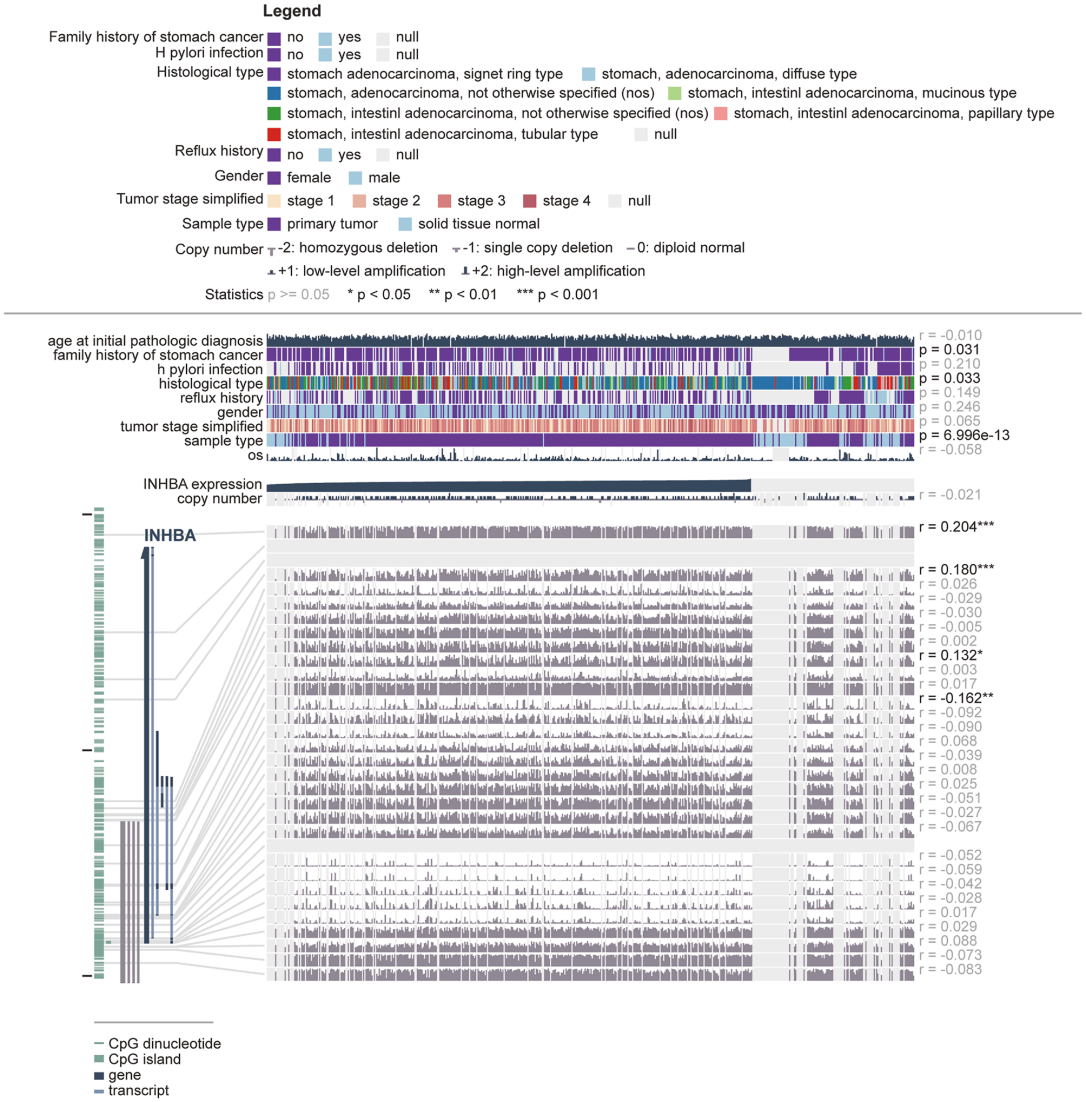
Visualization of the TCGA data for INHBA in gastric cancer using MEXPRESS database; the samples were arranged in order of their expression value. The view shows the correlation between INHBA expression and promoter region, clinical features, and CNVs, with the Pearson correlation coefficients on the right side. The height of the dark green line represents the INHBA expression value (normalized RNASeqV2 in TCGA) and the beta value for the Infinium 450 k probes.

Previous studies have shown that DNA promoter methylation is a meaningful pattern that affects tumorigenesis^30–32^. To explore the correlation between INHBA expression and DNA methylation, methylation levels of INHBA in GC were determined using the MEXPRESS database. Figure 12 shows the default MEXPRESS plot for INHBA expression in GC samples sorted based on the INHBA expression value. The results showed significant changes in methylation levels between tumor and normal tissues, indicating that INHBA expression might not be controlled by DNA methylation.

### Functional enrichment analysis of genes co-expressed with GC

**Figure 13:**
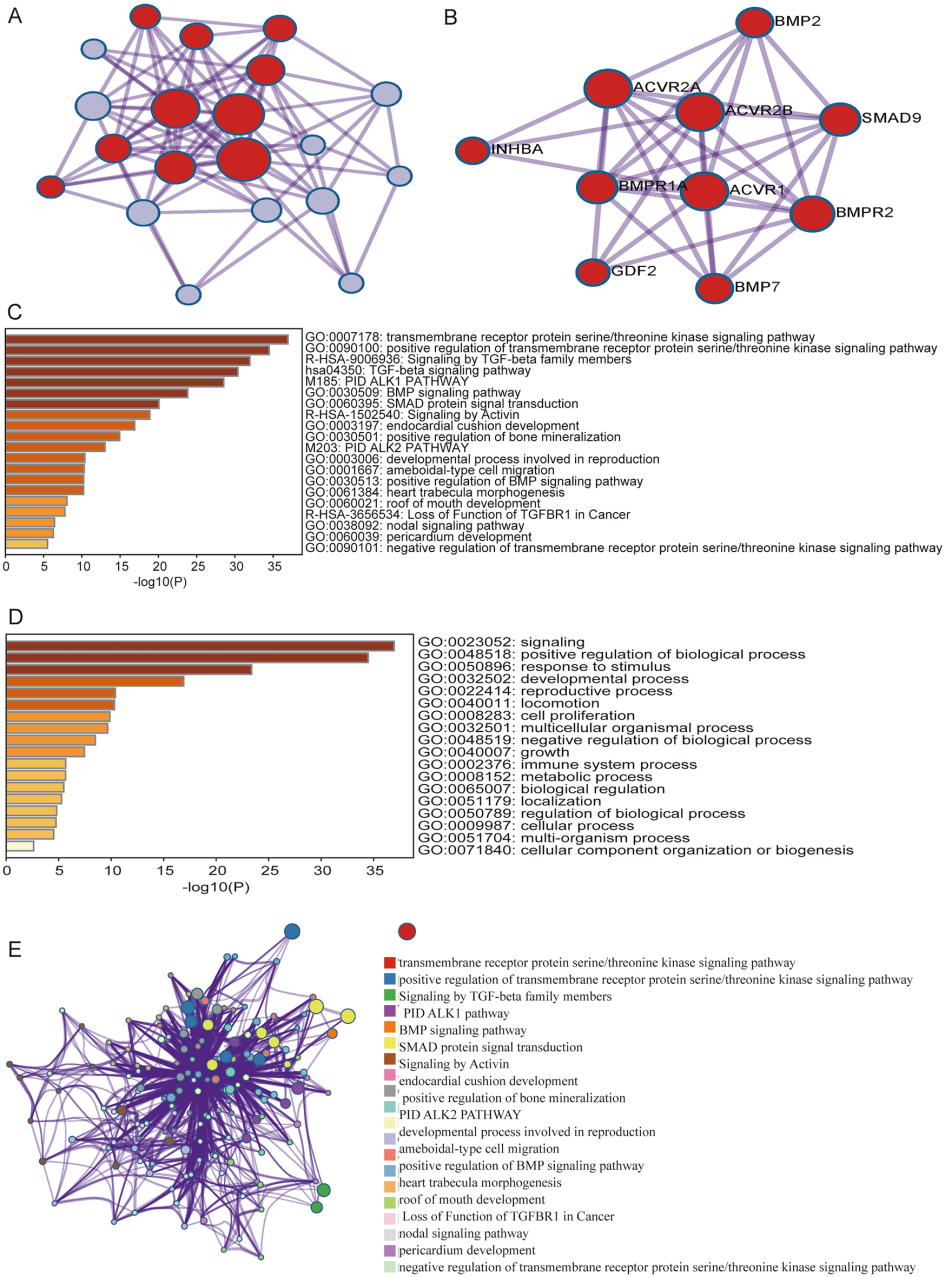
Protein–protein interaction network and functional enrichment analyses for INHBA (Metascape database). https://metascape.org/gp/index.html#/reportfinal/tyqpswf7z (**A**–**B**) Protein–protein interaction network and MCODE components identified in the gene lists. (**C**–**D**) Bar graph of enriched terms, colored based on *P*-values. (**E**) Network of enriched terms, colored by cluster ID, where nodes that share the same cluster ID are typically close to each other.

To determine the potential function of INHBA, we performed PPI network, GO function, and KEGG pathway enrichment analyses using the Metascape database. The PPI network is shown in Fig. 13A,B. As seen in Fig. 13C–E, the most significantly enriched GO terms were regulation of transmembrane receptor protein serine/threonine kinase signaling pathway, BMP signaling pathway, SMAD protein signal transduction, nodal signaling pathway, cell proliferation, and metabolic process. The most enriched KEGG pathways were TGF-β signaling and PID ALK1/2 pathways.

### GO function and KEGG pathway enrichment analyses of co-expression genes correlated with INHBA expression in GC

**Figure 14:**
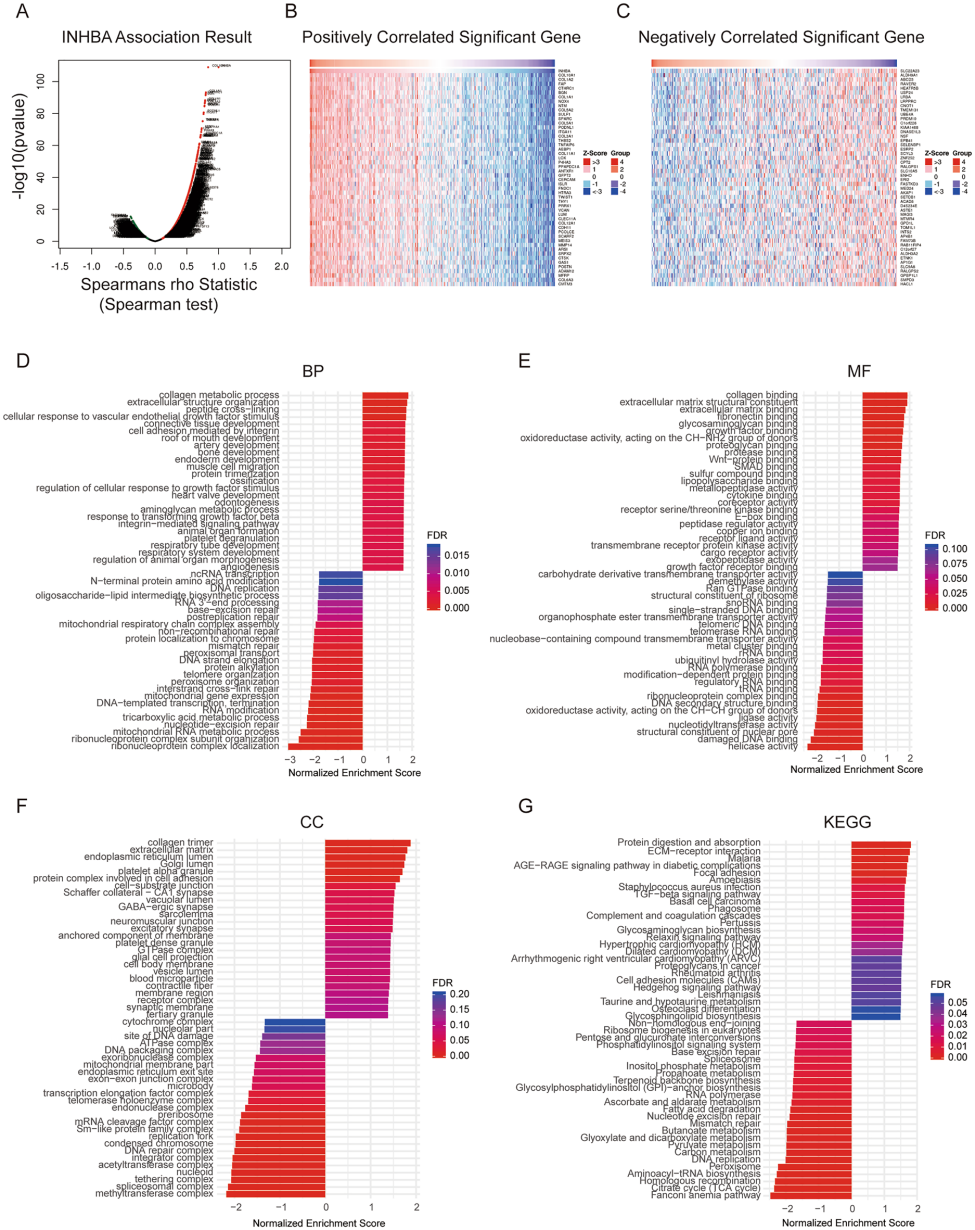
Genes differentially expressed with respect to INHBA in gastric cancer and the corresponding functional enrichment analysis (LinkedOmics database). https://gdac.broadinstitute.org/runs/stddata__2016_01_28/data/STAD/20160128/. (**A**) Volcano plot of correlation between INHBA and genes differentially expressed in GC, as assessed using T-test. (**B**–**C**) Heatmaps showing genes positively and negatively correlated with respect to INHBA in GC. Positively correlated genes have been indicated in red, while negatively correlated genes have been indicated in blue. (**D**) Biological processes. (**E**) Molecular function. (**F**) Cellular components. (**G**) KEGG pathway analysis. http://www.kegg.jp/kegg/kegg1.html.

LinkedOmics was used to obtain mRNA sequencing information from patients with GC in the TCGA-STAD cohort. Spearman’s test was conducted to analyze the correlations between INHBA and the genes that are differentially expressed in GC (red represents positively related genes, while green represents negatively related genes) (Fig. 14A). The Top 50 genes that were positively and negatively correlated with INHBA are shown in the heat maps (Fig. 14B,C). Importantly, GO and KEGG functional enrichment analyses conducted using GSEA suggested that these genes differentially expressed in correlation with INHBA in GC were mainly enriched in the biological processes of collagen metabolic process, extracellular structure organization, cellular response to vascular endothelial growth factor stimulus, connective tissue development, DNA replication, and so on (Fig. 14D). Essentially, the MF and CC were collagen binding, extracellular matrix structural constituent, growth factor, Wnt-protein binding, SMAD binding, collagen trimer, and endoplasmic reticulum lumen (Fig. 14E,F). KEGG pathway analysis showed that cancer-related signaling pathways were enriched, including the TGF-β, ECM-receptor, and AGE-RAGE signaling pathways (Fig. 14G).

### scRNA-seq analysis of INHBA expression in GC

**Figure 15:**
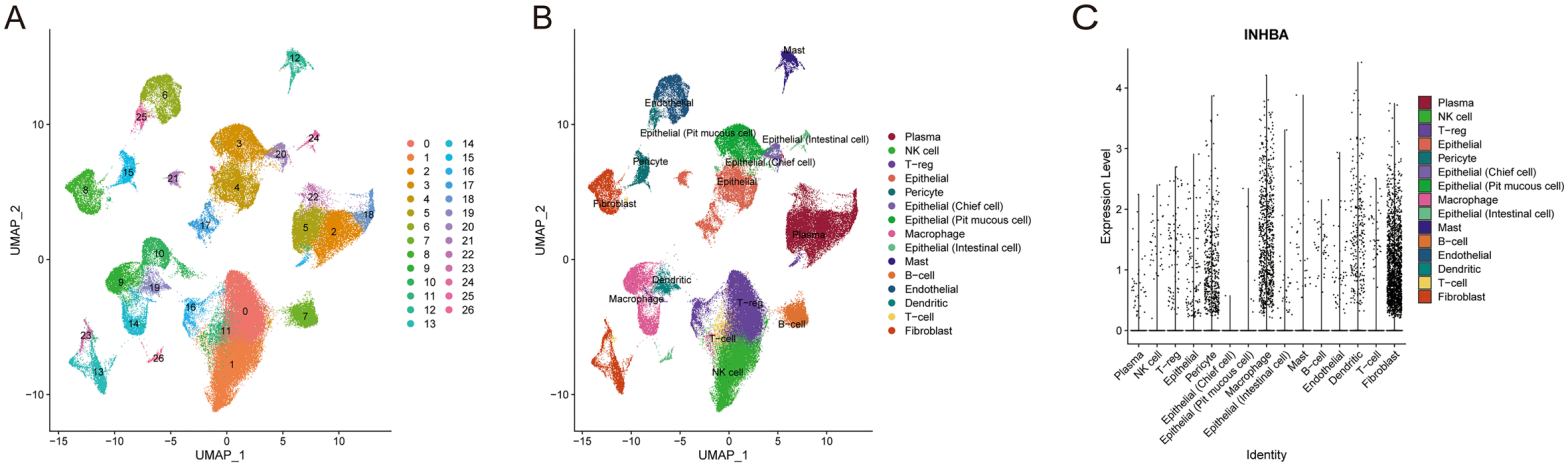
Single-cell data analysis based on 26 gastric cancer patients from GSE183904. (**A**) All cells from gastric cancer tissue were classified into 26 clusters. (**B**) The cell types were identified by tissue type-specific canonical markers defined in the literature. (**C**) The violin plot shows that INHBA is more highly expressed in fibroblast and pericyte than in other cell clusters.

The "ScaleData" function was used to scale all genes extracted from 26 primary gastric tumor tissues of the scRNA-seq dataset GSE183904 and performed PCA to reduce the dimension. Then, UMAP method was used for further dimension reduction, total cells were divided into 26 clusters (Fig. 15A). These identified cell clusters were labeled as different cell types. Type-specific canonical markers of tissue type are defined from the literature^33^, including myeloid, lymphoid, plasma, epithelial, and stromal cells (Fig. 15B). Notably, INHBA was more significantly expressed in fibroblasts and pericyte than in other cell clusters (Fig. 15C). Meanwhile, featurePlot revealed the expression of INHBA in all cell clusters (Fig. S1). Information of the cell subsets was presented in Table S1.

### The status of INHBA expression in GC MGC-823

**Figure 16:**
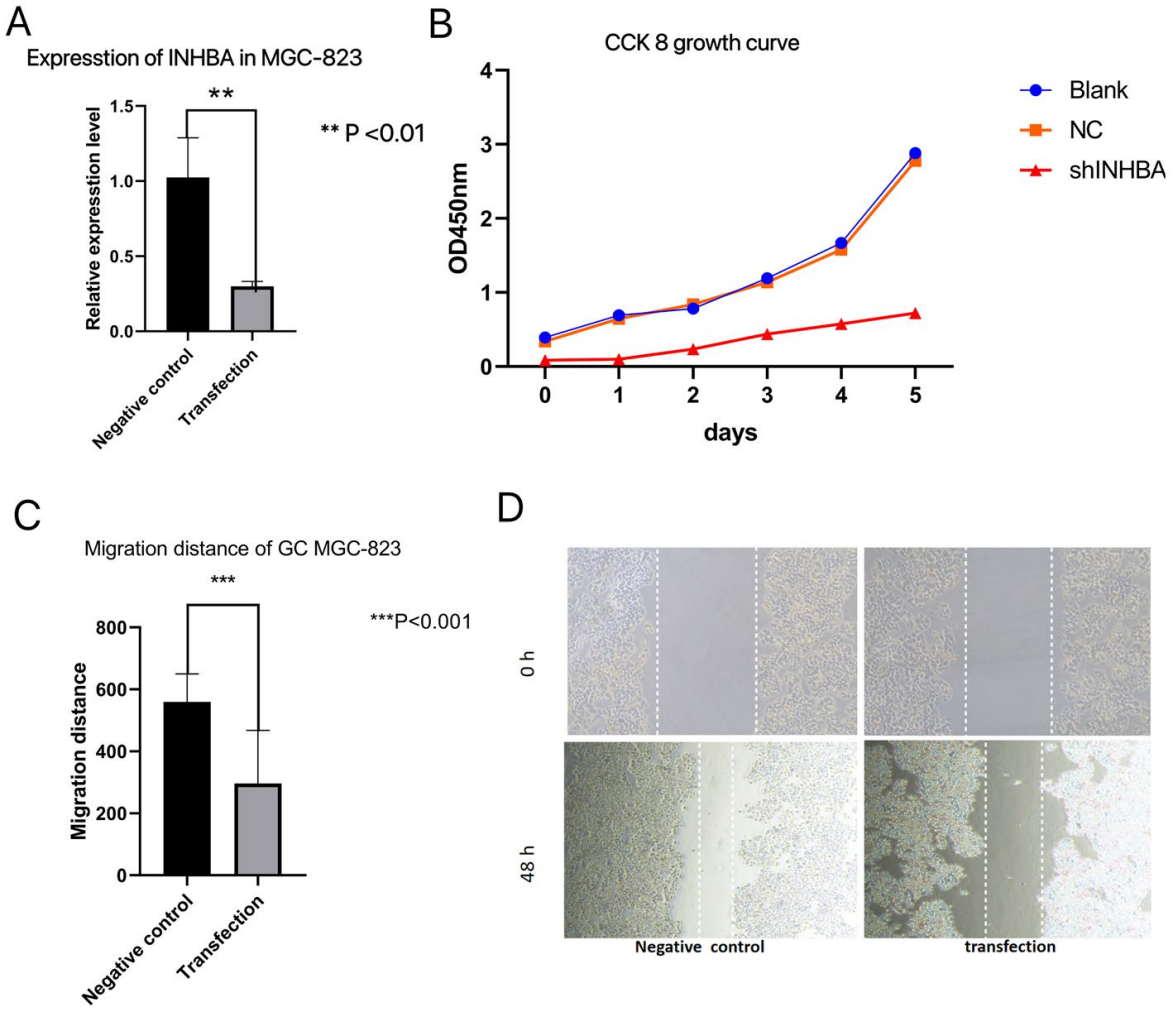
shINHBA inhibits migration and proliferation abilities of GC cells. (**A**) The expression level of GC MGC-823 after transfection with INHBA was determined by PCR. (**B**) CCK 8 growth curve to evaluate GC MGC-823 proliferation in the blank, control and transfection groups. (**C**) Migration distance of GC MGC-823 transfected with INHBA. (**D**) Images of cell migration in each group tested by scratch test.

The INHBA expression levels in the negative control and transfection groups were determined by real-time PCR, which showed that the expression levels of INHBA in the GC MGC-823 negative control group significantly exceeded the plasmid 0 transfection group (p < 0.05; Fig. 16A).

### shINHBA inhibits migration and proliferation abilities of GC cells

CCK-8 assay showed that there was no significant difference in cell viability between the blank group and NC group (p > 0.05). The viability of cells in the shINHBA group was significantly attenuated relative to the NC and blank groups (p < 0.05; Fig. 16B). Cell migration was measured by scratch assay, and the results showed that the transfected group migrated significantly less well compared to the negative control group (Fig. 16C,D, p < 0.05). These findings provide evidence that INHBA gene silencing suppresses migration, invasion, and proliferation in GC tissues.

## Discussion

GC is a heterogeneous malignancy with a high probability of recurrence and unfavorable prognosis^8,34^. Currently, mechanistic analysis have led to the identification of effective molecules for GC diagnosis and therapy, such as CASC2^35^, PROX1^36^, HOXA11^37^, circLMTK2^38^ and VCAM-AS1^39^. However, the 5-year survival rate of patients with GC remains unsatisfactory. Therefore, it is imperative to identify promising biomarkers and therapeutic targets for GC diagnosis and treatment. As an important member of the TGF-β family, INHBA is strongly associated with tumorigenesis and progression in multiple types of solid tumors, such as de novo acute myeloid leukemia^40^, non-small cell lung cancer (NSCLC)^41^, colorectal cancer (CRC)^42–44^, esophageal cancer^45^, prostate cancer (PC)^46^ and ductal carcinoma^47^. However, the specific role of INHBA in GC remains unknown.

In this study, changes in INHBA mRNA expression in GC and the expression status of INHBA in different tumor subgroups were investigated. The results revealed that the INHBA mRNA expression level was higher in GC than in normal gastric mucosa tissue, consistent with the results of previously published studies^23,25,26^. Validation experiments carried out by means of IHC and qRT-PCR also revealed that the protein expression level of INHBA was high in GC tumor tissues. Elevation of INHBA has also been reported in adenocarcinoma, NSCLC, CRC, and PC^14,41,43,44,46^. This strongly indicates that INHBA plays a critical role in tumorigenesis and progression. Previous studies have found that high INHBA expression is associated with methylation in some tumors^14,41,43,44^. We utilized the MEXPRESS database to uncover the methylation levels of INHBA in GC, but the results only revealed weak significant methylation level changes between tumor and normal tissues, suggesting that INHBA expression might not be controlled mainly by promoter methylation in GC, as seen in case of other tumors. Relevant studies have shown that there is a BHHE40 binding site near the INHBA promoter region, and the transcription factor BHLHE40 directly regulates the high expression of INHBA, thus promoting the proliferation and migration of colon cancer cells^48^. Other studies have shown that DNA amplification plays a key role in the upregulation of INHBA in patients with head and neck squamous cell carcinoma (HNSC). Further analysis shows that INHBA expression is negatively correlated with DNA methylation, and INHBA is significantly negatively correlated with the regulation of miR-217-5p expression. This is consistent with the regulatory relationship between mirna and target genes^49^. The mechanism underlying elevated INHBA expression in GC requires further research.

Seder et al*.* reported that INHBA is upregulated in LUAD, and its overexpression is associated with worse survival in stage I adenocarcinoma^14^. Studies by Okano et al*.* showed that INHBA is was significantly upregulated in CRC and that INHBA could serve as a useful predictive biomarker for the prognosis of CRC^42,43^; the same result was reported by Liu et al*.* in ductal carcinoma^47^. Our research showed that high expression levels of INHBA in GC were significantly related to unfavorable prognosis, especially in the clinicopathological subtypes of male, HER2-positive, and intestinal classification. In the subgroup analysis, we found that compared with the normal group, the subgroup with TP53 mutation had relatively higher INHBA expression, and the difference was statistically significant, suggesting that TP53 mutation was correlated with INHBA expression. Meanwhile, INHBA has high diagnostic value for distinguishing GC patients from healthy individuals. Thus, it is reasonable to believe that INHBA may serve as a potential prognostic and diagnostic biomarker for GC, especially in case of specific subtypes.

Tumor infiltration is significantly associated with the progression and prognosis of tumors^27–29^. Recently, Kumar et al*.* found that there was a strong correlation between FAP and INHBA co-expression levels, both of which showed higher expression in tumor fibroblasts than in normal tissues. They also reported an increased proportion of plasma cells in diffuse-type GC^50^. We used TIMER and ssGSEA methods to identify the relationship between INHBA expression levels and immune infiltration. The results showed that INHBA was negatively correlated with B cells and positively correlated with macrophage, neutrophil, and dendritic cell infiltrations. The above results indicated that INHBA is involved in tumor infiltration. We also found that INHBA in GC to be positively correlated with CD8A expression in CD8+ T cells, and MPO, FCGR3B, FPR1, and CSF3R expression in neutrophils. Similarly, INHBA expression in GC was positively correlated with CD68, CD84, CD163, and MS4A4A expression in macrophages and CD209 expression in dendritic cells. The cell phenotype mentioned above plays a significant role in immune infiltration, which further confirms that INHBA in GC is correlated with immune infiltration. Prognostic analysis of INHBA expression levels in GC, based on immune cell subgroups, revealed that high expression of INHBA in GC had a worse prognosis in enriched B cells, CD4+ T cells, CD8+ T cells, and macrophages. These results strongly suggested that high INHBA expression in GC may affect prognosis, partly because of immune infiltration. In addition, INHBA was found to be significantly positively correlated with most steps of the cancer immunity cycle and ImmuneScore, StromalScore, and ESTIMATEScore, which strongly indicated that INHBA would be a promising predictor of immunotherapy response, with a higher INHBA indicating greater sensitivity. Moreover, we used multiple approaches to identify dasatinib, PI-103, ML210, ML162, mk-2461, temsirolimus, romidepsin, YM-155, and LY2606368 as promising therapeutic drugs for INHBA-high patients with GC.

To further explore the potential mechanism of action of INHBA in regulating the progression and immune microenvironment in GC, genes co-expressed with INHBA in GC were examined. The results showed that INHBA mainly enriched cancer-related signaling pathways, including the TGF-β, ECM-receptor, PID ALK1/2, and AGE-RAGE signaling pathways. In addition, INHBA gene silencing inhibited GC progression by inactivating the TGF-β pathway. INHBA expression was elevated in GC and activated the migration, invasion, and proliferation abilities of GC cells, consistent with the results of Chen et al.^51^.

To summarize, we found that the expression level of INHBA was associated with prognosis of GC, and INHBA could serve as a promising predictor of immunotherapy response. INHBA may affect the prognosis of GC through a new mechanism, immune infiltration, which may provide new insights for future in-depth studies. However, this study also has some limitations. Due to the limitation of the database, we did not continue to explore the deep correlation between INHBA and immune infiltration. In addition, there is an urgent need to carry out further experiments to verify the results of this study, which we plan to do in many related studies in the future.

## Materials and methods

### Tissue collection

All fresh specimens were collected between January 2018 and June 2019 at Hebei Medical University Fourth Affiliated Hospital. Resected GC tissues and matched adjacent non-tumor gastric tissues (n = 65) were snap-frozen in liquid nitrogen and stored at − 80 °C for quantitative real-time PCR (qRT-PCR) assay. None of the patients was treated with radiotherapy or chemotherapy before surgery.

### RNA extraction and qRT-PCR

RNA was extracted according to the manufacturer’s instructions. Primers were purchased from GeneCopoeia (catalog number: HQP017978, Eockville, MD, USA). INHBA sequences were as followed: Forward: 5’-CAT TGC TCC CTC TGG CTA TCA T-3', Reverse: 5'-GCA CAC AGC ACG ATT TGA GGT T-3'. The housekeeping gene *GAPDH* (catalog number: ab9485; bioWORLD, USA) served as an interval reference to normalize the RNA abundance. The *GAPDH* sequences were as followed: Forward: 5'-GCA CCG TCA AGG CTG AGA AC-3', Reverse: 5'-ATG GTG GTG AAG ACG CCA GT-3'. The relative FCs in mRNA expression were expressed in terms of geometric mean and calculated using the 2^−ΔCT^ method. All reactions were conducted in triplicate, and the results are represented as mean ± standard deviation.

### Immunohistochemistry (IHC)

INHBA expression in tumors and adjacent normal tissues were detected using IHC. The anti-INHBA monoclonal antibody was purchased from ABclonal (used at a dilution of 1:60; catalog number: a6614; ABclonal, Wuhan, China). All slides were independently interpreted by two pathologists who were blinded to clinical information. The IHC staining score included the proportion of positively stained tumor cells and staining intensity. The proportion of positively stained tumor cells was scored as follows: 0 (no tumor cells stained), 1 (< 25% tumor cells stained), 2 (25%–50% tumor cells stained), 3 (50%–75% tumor cells stained), and 4 (75%–100% tumor cells stained). Staining intensity was graded using the following criteria: 3 (brown, strong staining), 2 (yellow brown, moderate staining), 1 (light yellow, weak staining), and 0 (no staining). The final total staining score was calculated by multiplying the proportion of stained tumor cells by the staining intensity score. INHBA expression was scored; a score of ≤ 3 indicated negative INHBA expression, while a score of > 3 indicated positive INHBA expression. A score of ≤ 6 indicated low expression, while a score of > 6 indicated high expression. Pathological diagnosis was made in accordance with the histological classification of tumors developed by the World Health Organization.

### Oncomine database analysis

Oncomine (www.oncomine.org) database, which contains 715 gene expression datasets as well as 867,33 cancers and normal samples, is the biggest and user-friendly oncogene chip database and integrated data mining tool^52^. DNA copy number and mRNA expression differences of the INHBA gene between GC tumors and normal tissues were determined using the Oncomine database. In the present study, we incorporated samples from a series of GC studies, including Cho GC^53^, DErrico GC^54^, Deng GC^55^, Cui GC^56^, Chen GC^57^, TCGA GC^58^, and Wang GC^59^. The expression of INHBA in GC tissues was evaluated with respect to its expression in normal tissues; results were considered statistically significant at *P* < 0.05, with a fold-change (FC) of 1 used as the cut-off criterion.

### GEPIA2 database analysis

GEPIA2 (http://gepia2.cancer-pku.cn/) is an updated version of GEPIA for analyzing the transcript data of 198,619 isoforms and 84 cancer subtypes, including 9,736 tumor samples and 8,587 normal tissue samples from TCGA and Genotype-Tissue Expression projects, using a standard processing pipeline^60,61^. In the present study, we used GEPIA2 to analyze the expression levels of INHBA in GC tumor and normal tissues.

### UALCAN database analysis

UALCAN (http://ualcan.path.uab.edu/) is a user-friendly web resource for analyzing cancer transcriptome data and in-depth gene expression, methylation information, and survival curves^62^. In the present study, we used UALCAN to evaluate INHBA expression in different tumor subgroups, such as tumor stage, grade, nodal metastasis status, sex, TP53 mutation status, race, age, historical subtypes, and HPV status.

### TIMER database analysis

TIMER (https://cistrome.shinyapps.io/timer/) is a comprehensive and user-friendly online tool for systematically investigating and visualizing the correlation between immune infiltrates and a wide spectrum of factors, including gene expression, clinical outcomes, and somatic mutations, in over 10,897 tumors from 32 cancer types^63,64^. The differential expression of INHBA between tumor and normal tissues was evaluated using the Diff Exp module across all TCGA database tumors, the results for which are shown as boxplots. The abundances of six immune infiltrates (CD8+, T cells, B cells, CD4+ T cells, macrophages, neutrophils, and dendritic cells) were assessed by means of a statistical method. In addition, we also analyzed the comparison of tumor infiltration levels among tumors with different somatic copy number alterations for INHBA in GC. Statistical significance was set at *P* < 0.05.

### Kaplan–Meier plotter database

Receiver operating characteristic (ROC) curves were obtained using the pROC^65^ package in R software, to explore the sensitivity and specificity for distinguishing GC patients from healthy individuals. The Kaplan–Meier plotter (http://kmplot.com/) is an online database containing microarray gene expression data and survival information extracted from the GEO and TCGA databases, which contain gene expression data and survival data of 1,065 GC patients^66^. A valid number of GC patients were included in this study, after excluding patients with missing expression values or those who did not have complete clinical data, including survival time and status. In order to investigate the underlying prognostic value of INHBA, we evaluated overall survival (OS), first progression survival (FPS), and post-progression survival (PPS) using the Kaplan–Meier plotter database, based on median expression (high *vs.* low), and then assessed them using Kaplan–Meier survival plots, in terms of hazard ratio (HR) with 95% confidence intervals (95% CI) and log rank *P*-value. Furthermore, the correlations between INHBA expression and different types of clinicopathological characteristics, such as sex, human epidermal growth factor receptor 2 (HER2) status, and Lauren classification, were also analyzed using this database. Correlation between INHBA expression and prognosis in GC patients with enriched immune infiltrates were also evaluated using the Kaplan–Meier plotter database.

### cBioportal database

cBioportal (http://www.cbioportal.org/) is an interactive open-source platform that contains 245 cancer studies, which provides large-scale cancer genomics datasets to visualize, analyze, and download^67,68^. The frequency of INHBA alterations (amplification, deep deletion, and missense mutations) in patients with GC was assessed using the cBioportal for Cancer Genomics database and TCGA. Furthermore, we used Kaplan–Meier analysis in cBioportal to analyze the effect of INHBA expression dysregulation on the OS and disease-free survival (DFS) of patients.

### MEXPRESS database analysis

MEXPRESS (https://mexpress.be/) is an intuitive and user-friendly online web tool for the visualization of TCGA gene expression (normalized RNASeqV2 value), DNA methylation, and clinical data, as well as the correlation between them for a single gene of interest^69,70^. By default, the samples were sorted on the basis of the expression (from low to high) of the gene of interest. Pearson’s correlation was used to calculate the difference between expression values and methylation data. In this study, we evaluated the correlation between INHBA expression and promoter methylation in GC.

### Functional enrichment analysis

Metascape (http://metascape.org) is a new, free, and user-friendly gene list analysis tool for functional enrichment analysis, which includes cellular component (CC), biological process (BP), molecular function (MF), KEGG^71–73^ pathway, and protein–protein interaction (PPI) analyses^74^. In the present study, Metascape was used to perform GO and KEGG pathway analyses of INHBA and neighboring genes significantly associated with INHBA. Statistical significance was set at *P* < 0.05.

### LinkedOmics analysis

The LinkedOmics database (http://www.linkedomics.orglogin.php) is a comprehensive and unique online tool for accessing, analyzing, and comparing disseminating data from large-scale cancer omics projects, within and across all 32 TCGA cancer types^75^. Three LinkedOmics analytical modules were applied to explore attributes that are associated with the gene of interest, perform functional enrichment analysis, and compare integrated association results. In the present study, we used this database to explore the differentially expressed genes related to INHBA in the TCGA stomach adenocarcinoma (STAD) cohort. GSEA was used to perform GO and KEGG pathway analyses.

### Immune microenvironment and INHBA

The infiltration levels of immune cell types in the tumor microenvironment (TME) were quantified using the ssGSEA method, and characteristic marker genes for each immune cell type were selected based on published literature^76^. The ImmuneScore and StromalScore for every GC patient were calculated using the ESTIMATE algorithm based on “estimate” package, which takes advantage of the unique properties of mRNA expression, to infer tumor cellularity and purity^77^.

Drug sensitivity data of cancer cell lines were obtained from the Cancer Therapeutics Response Portal (CTRP)^78^ and PRISM^79^ databases. Both provide the area under the dose–response curve (AUC) as a measure of drug sensitivity. Lower AUC values indicate an increased sensitivity to therapy. K-nearest neighbor (K-NN) imputation was applied to impute the missing AUC values. A ridge regression model was applied to obtain a drug sensitivity estimate between the two datasets for every GC patient. Expression profiles of human cancer cell lines were obtained from the Broad Institute Cancer Cell Line Encyclopedia database (https://sites.broadinstitute.org/ccle)^80^.

### scRNA-seq analysis

The gastric cancer scRNA-seq datasets GSE183904 was downloaded from Gene Expression Omnibus (GEO; https://www.ncbi.nlm.nih.gov/geo/) which included 29 gastric cancer tissues and 11 normal tissues. We focused on 26 of primary gastric tumor tissues and performed a follow-up analysis. Finally, a total of 115,792 cells were included in the original data.

The percentage of mitochondria and rRNA was calculated by the PercentageFeatureSet function for the gene expressing > 500& < 6000. the mitochondrial content was < 20%. Normalized the merged data and find the top 2000 highly variable genes by the "FindVariableFeatures" function (based on variance-stabilized transformation ("vst") to identify variable features). Meanwhile, the "ScaleData" and "RunPCA" functions were used to reduce the dimension of the the first 2000 highly variant genes. Next, the "RunUMAP" function with dims = 30 was used to perform Uniform Manifold Approximation and Projection (UMAP) method to further reduce the dimension and visualize merged data. Then, we clustered cells by "FindNeighbors" and "FindClusters" functions (resolution = 0.5). Finally, featureplot and violin plots were used to visualize the expression of INHBA between different cell clusters.

### Statistical analysis

R (version 3.6.5, USA) and Prism (version 8, GraphPad Software, La Jolla, CA, USA) were used for statistical analysis in this study. Continuous variables were analyzed using the Student’s *t*-test. Statistical significance was set at *P* < 0.05.

### Plasmid transfection

Cells in the log growth phase were selected for the experiments. The collected cell suspensions were seeded in 6-well plates with 5104 cells/well per well. Cells were cultured in complete medium until the cells reached 50–80% confluence. Liposome-mediated transfection was performed according to the instructions of the liposome 2000 manufacturer (11,668–027; Invitrogen Life Technologies). Cells were divided into groups of blank (human GC MGC-823 cells transfected with empty plasmid), negative control (NC; human GC MGC-823 cells transfected with shRNA sequence), shINHBA-1 (human GC MGC-823 cells transfected with shINHBA-1), shINHBA-2 (human GC MGC-823 cells transfected with shINHBA-2), and shINHBA-3 (human GC MGC-823 cells transfected with shINHBA-3). Before transfection, cells in the log growth phase were seeded in 6-well plates. When cell confluence reached 90–95%, cells were transferred to serum-free opti-minimum essential medium (Opti-MEM; Gibco, Carlsbad, CA) for culture. Transfections were then performed according to the lipossome 2000 specification.

### Cell Counting Kit‐8 (CCK‐8) assay

The gastric cancer cell lines 48 h after plasmid transfection were digested and evenly seeded in 2 × 103 in 96-well plates. Five complex wells were set for each group in three groups, namely blank, control and transfection groups. The outermost peripheral bore of the 96-well plate was filled with sterile PBS to prevent edge effects. After 4 h, 100 μl of complete cell culture per well was added with 10μlCCK8 of reagent to prepare the mixed culture medium. The corresponding cell culture wells were also replaced and incubated in a cell culture incubator for 2 h. The 96-well plate was placed in Cytation5, and the absorptive value at 450 nm was detected on a multi-function microplate reader for 5 days, and recorded the absorptive value and then draw the CCK 8 growth curve.

### Scratch test

Following a 48‐hr transfection period, When cell confluence reached about 90%, the central axis of the well was gently scratched with a sterile pipette tip. After PBS washing removed the floating cells, serum‐free medium was added to restore the cells for 0.5–1 h. Following cell recovery, the cells were photographed from 0 to 48 h, and the cell migration distance was calculated by randomly taking 12 lines analysis using Image J.

### Ethical statement

(1) The study was conducted in accordance with the Declaration of Helsinki, and approved by the Institutional Review Board (or Ethics Committee) of Ethics Committee of the Fourth Hospital of Hebei Medical University (protocol code 2018MEC108 and October 2018) of approval for studies involving humans. (2) Confirms that all experiments were performed in accordance with relevant named guidelines and regulations. (3) Confirms that informed consent was obtained from all participants and/or their legal guardians.

### Consent

Written informed consent was obtained from the individual or guardian.

## Supplementary Information

Supplementary Information.

## Data Availability

Data are available upon request from corresponding author Guangjie Liu.

## Abbreviations

INHBA: Inhibin subunit beta A
TGF-β: Growth factor-beta
GC: Gastric cancer
TIMER: Tumor Immune Estimation Resource
GEPIA2: Profiling Interactive Analysis
GEO: Gene Expression Omnibus
GO: Gene Ontology (GO)
KEGG: Kyoto Encyclopedia of Genes and Genomes
LUAD: Lung adenocarcinoma
qRT-PCR: Quantitative real-time PCR
OS: Overall survival
FPS: First progression survival
PPS: Post-Progression Survival
IHC: Immunohistochemistry
DFS: Disease-free survival
CC: Cellular component
BP: Biological process
MF: Molecular function
PPI: Protein–protein interaction
STAD: Stomach adenocarcinoma
TME: Tumor microenvironment
CTRP: Cancer Therapeutics Response Portal
AUC: Area under the dose–response curve
K-NN: K-nearest neighbor
