# Supplementary material for: Identification of INHBA as a potential biomarker for gastric cancer through a comprehensive analysis
Source: Sci Rep. 2023 Aug 1;13:12494. doi: 10.1038/s41598-023-39784-1 (PMC10394090; doi:10.1038/s41598-023-39784-1)
Supplement: Supplementary file 1 — Supplementary Information. [file 41598_2023_39784_MOESM1_ESM.pdf]

Figure S1

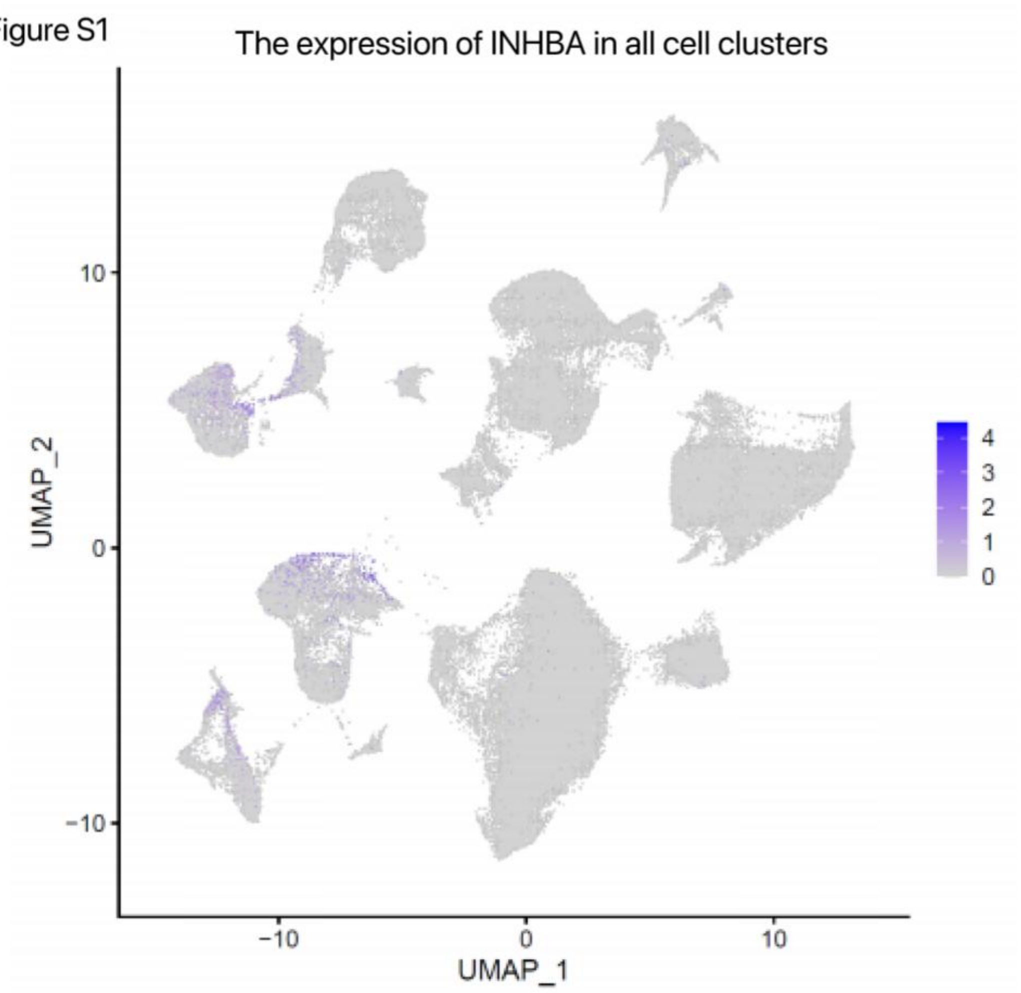

Table S1 The information of 26 cells types

**Cluster**	**Cell type annotation**
0	T-reg
1	NK cell
2	Plasma
3	Epithelial (Pit mucous cell)
4	Epithelial
5	Plasma

6	Endothelial
7	B-cell
8	Fibroblast
9	Macrophage
10	Unknown
11	T-cell
12	Mast
13	Fibroblast
14	Macrophage
15	Pericyte
16	T-reg
17	Epithelial
18	Plasma
19	Dendritic
20	Epithelial (Chief cell)
21	Epithelial
22	Plasma
23	Fibroblast
24	Epithelial (Intestinal cell)
25	Pericyte
26	Epithelial (Intestinal cell)
